# Studies on Mapping Plant Genes That Confer Tolerance to Abiotic Stresses

**DOI:** 10.3390/ijms231810760

**Published:** 2022-09-15

**Authors:** Richard R.-C. Wang

**Affiliations:** USDA-ARS Forage and Range Research Laboratory, Utah State University, Logan, UT 84322-6300, USA; richard.wang@usda.gov

Climate change is affecting the Earth’s environment through temperature fluctuation, rainfall patterns, wind, and radiation. These environmental conditions can cause abiotic stresses (heat, cold, drought, salinity, waterlogging, UV, and heavy metals, etc.) on plants. Plants under abiotic stress can be stunted, leading to yield loss in biomass and/or seed. To produce sufficient plant products for the future demand of food and feed, plant breeders are striving to improve the plant tolerance to multiple abiotic stresses. Therefore, knowledge on stress tolerance mechanisms, gene functions, and locations has become increasingly critical. Thus, the Special Issue “Mapping Abiotic Stress-Tolerant Genes in Plants” of the International Journal of Molecular Sciences (*IJMS*) was launched in 2019–2020, resulting in the publication of a collection of relevant papers [1].

Because of the importance of this topic, more researchers are devoting their academic efforts to studying genes that confer tolerance to abiotic stresses in plants. Whole-genome sequencing techniques have been widely used in many plant species, allowing the precise localization of genes to chromosomes. Mapping genes for abiotic-stress-tolerant genes to chromosomes is helpful to breeders in produce resilient crops to sustain crop production. Therefore, the *IJMS* Special Issue was continued for the year 2020–2021.

In this 2021 Special Issue, there were seven research articles [2,3,4,5,6,7,8] and one review article [9]. The seven research articles reported on studies on the downregulated-associated proteins (DrAPs), Expansin (EXP), Benzoxazinoids (BXs), domain of unknown function (DUF), PIN-FORMED (PIN), YUCCA flavin monooxygenases (YUC), Glycoside Hydrolase 3 (GH3), and UDP-glucuronosyltransferases (UGTs), SIMILAR TO RCD-ONEs (SROs), and Trehalose-6-Phosphate Synthetase (TPS) gene families (Table 1). The review article [9] examined the effects of abiotic stresses on parasitic plants and, in turn, its host.

Genes for downregulated-associated proteins in wheat, TaDrAp1 (TaNC2α1, TaNF-YC6 = TC233433) and TaDrAp2 (TaNC2α2, TaNF-YC8 = TC241235), were identified, and their expression and genetic polymorphism were studied by Zotova et al. [2]. Each of these two genes has three homeologs attributable to the A, B, and D genomes of hexaploid wheat. The homeologs of TaDrAp1 (TaDrAp1-A4, -B4, and -D4) are proximally located on the wheat chromosomes of homoeologous group 4, whereas those of the TaDrAp2 (TaDrAp2-A1, -B1, and -D1) are distally on group 1 (Table 1). The former gene was downregulated by drought and the latter was upregulated, indicating that the latter ones responded positively to abiotic stress. As I opened a Special Topic collection entitled “Mapping Essential Genes and Adaptation Genes Based on Genome Assembly and Annotation of Plants and Animals” in *IJMS* to test the hypothesis that essential genes for growth and development are proximally located while adaptation genes are distally located, it would be interesting to investigate whether TaDrAp1 controls the development and growth of wheat, such as the flowering time, plant height, and seed productivity, and whether TaDrAp2 is involved in drought tolerance.

The expansin gene family of moso bamboo is composed of four subfamilies, alpha-expansin, beta-expansin, epansin-like A, and expansin-like B, which have 45, 29, 7, and 1 genes, respectively [3]. These 82 genes were located to 32 scaffolds, instead of chromosomes, because the whole-genome sequencing work has not linked the scaffolds to chromosomes yet. Overall, there were 37 proximally located and 45 distally located genes. This suggests that many of the 82 expansin or expansin-like genes are not directly responsible for tolerance to abiotic stresses. Further research on the gene function of the individual genes is needed.

Benzoxazinoids (BXs) of rye (*Secale cereale* L.) could protect plants against nematodes and weeds through allelopathy [4]. However, there was no reported information on the number of genes and their chromosomal locations. DUF569 (AT1G69890) of approximal 3600 domain of unknown function (DUF) genes enhanced drought tolerance in Arabidopsis thaliana by positively regulating the production of ABA, polyphenols, flavonoids, carotenoids, and chlorophylls. This gene was distally located on chromosome 1 [5]. In A. thaliana, 10 genes (TAR2, At4g24670; YUC5, At5g43890; IAR3, At1g51760; GH3.1, At2g14960; GH3.3, At2g23170; GH3.12, At5g13320; UGT74E2, At1g05680; DAO2, At1g14120, PIN2, At5g57090, and PIN4, At2g01420) were involved in responses to salt and mannitol treatment [6]. These 10 genes disrupt auxin biosynthesis and were found on chromosomes 1, 2, 4, and 5 with the 5:5 proximal to distal ratio. The SIMILAR TO RCD-ONEs (SROs) gene family in sesame has two subfamilies, SiSRO1 and SiSRO2, which have two genes each; but SiSRO2a performed its function by regulating defense response and hydrogen peroxide metabolic process, while SiSRO2b associated genes mainly participated in biological processes related to hormonal and stress responses [7]. Three of these four genes are located in linkage groups (LGs) of sesame; SiSRO1a on LG2, SiSRO1b and SiSRO2a on LG3, while SiSRO2b on a scaffold. Both genes on LG3 were distal but at the same distance from the opposite ends. In watermelon, the Trehalose-6-Phosphate Synthetase (TPS) gene family has seven genes (ClTPS1 to 7) that confer tolerance to salt and other stresses by maintaining osmotic pressure, protecting membrane structure, and participating in the signal transduction [8]. Trehalose upregulated the activities of antioxidant enzymes, such as superoxide dismutase (SOD), ascorbic acid peroxidase (APX), peroxidase (POD), and catalase (CAT); thus, increasing reactive oxygen species (ROS) scavenging capacity. All ClTPS genes, except ClTPS2, are distally located (Table 1).

Of the 109 genes mapped to chromosomes (or scaffolds), 47 were regarded as proximally located and 62 distally located (Table 1). The 1.32:1 distal/proximal ratio for genes reported in this 2021 Special Issue is lower than that determined in the 2020 issue, 2.20:1 [10]. It is possible that more of the genes studied in this 2021 Special Issue are involved in essential biological pathways that control plant growth and development rather than in those functions that directly respond to abiotic stresses.

In conclusion, plant genes that confer tolerance to abiotic stresses tend to be distally located on physical chromosomes. Their upstream promoter regions contain many cis-acting regulatory elements that respond to phytohormones, abiotic stresses, and biotic stresses. These plant genes are characterized by spatial–temporal expression. With knowledge of the gene locations and expression profiles, plant breeders can develop efficient strategies to utilize the available genes that confer tolerance to abiotic stresses caused by climate changes.

## Figures and Tables

**Table 1:** Research articles on gene families associated with tolerance to abiotic stresses in this Special Issue on mapping genes to chromosomes.

Plant Species	Gene Family	Gene/Gene Subfamily	No. of Genes	Stress(es)	Mechanism	Chromosome (Length in bp)	Position	Proximal (p) or Distal (d)	Reference
*Triticum aestivum* L. bread wheat	Downregulated-associated protein	*TaDrAp1* (TC233433)	3	drought	modulating flowering time, plant height, and seed productivity	4A (744,588,157)	306,315,444–306,320,631	0.41 = p	Zotova et al. 2020 [2]
						4B (673,617,499)	316,654,368–316,664,398	0.47 = p	
						4D (509,857,067)	220,464,822–220,469,159	0.43 = p	
		*TaDrAp2* (TC241235)	3			1A (594,102,056)	501,702,108–501,707,597	0.84 = d	
						1B (689,851,870)	546,528,780–546,533,951	0.79 = d	
						1D (495,453,186)	406,545,964–406,551,455	0.82 = d	
*Phyllostachys edulis* moso bamboo	Expansin	alpha-Expansin (*EXPA*)	45	drought	involved in cell wall loosening and cell enlargement; upregulated by abscisic acid (ABA) and polyethylene glycol (PEG) treatments	15 scaffolds	NA	22 p:23 d	Jin et al. 2020 [3]
		beta-Expansin (*EXPB*)	29			10 scaffolds	NA	12 p:17 d	
		expansin-like A (*EXLA*)	7			6 scaffolds	NA	2 p:5 d	
		expansin-like B (*EXLB*)	1			1 scaffold	NA	1 p:0 d	
*Secale cereale* rye	Benzoxazinoids (BXs)	*BX1, HBOA, GDIBOA, DIBOA, GDIMBOA, DIMBOA,* and *MBOA*	?	nematodes and weeds	allelopathy	NA	NA	NA	Rakoczy-Trojanowska et al. 2021 [4]
*Arabidopsis thaliana* Thale cress	domain of unknown function (DUF) genes	*DUF569 (AT1G69890)*	~3600	drought	positive regulator of production of ABA, polyphenols, flavonoids, carotenoids, and chlorophylls	1 (30,432,564)	26,323,086–26,324,946	0.86 = d	Nabi et al. 2021 [5]
*Arabidopsis thaliana* Thale cress	PIN-FORMED (PIN), YUCCA flavin monooxygenases (YUCs), Glycoside Hydrolase 3 (GH3), and UDP-glucuronosyl-transferases (UGT)	*TAR2, At4g24670; YUC5, At5g43890; IAR3, At1g51760; GH3.1, At2g14960; GH3.3, At2g23170; GH3.12, At5g13320; UGT74E2, At1g05680; DAO2, At1g14120, PIN2 (At5g57090)* and *PIN4 (At2g01420)*	10	salt and osmotic (mannitol)	disruption of auxin biosynthesis, but especially in the processes of amideand ester conjugation	chromosomes 1, 2, 4 and 5		Chr1—1p:2d Chr2—2p:1d Chr4—1 p Chr5—1 p:2 d	Smolko et al. 2021 [6]
*Sesamum indicum* sesame	SIMILAR TO RCD-ONEs (SROs)	*SiSRO1a*	4	osmotic, salt, cold, heat, and submerge	SiSRO2a perform its function by regulating defense response and hydrogen peroxide metabolic process, while SiSRO2b-associated genes mainly participated in biological processes related to hormonal and stress responses.	LG2 (~18.5 Mb)	6,894,030–6,901,957	0.35 = p	Liu et al. 2021 [7]
		*SiSRO1b*				LG3 (~25 Mb)	21,219,139–21,221,593	0.84 = d	
		*SiSRO2a*				LG3 (~25 Mb)	3,993,893–3,996,925	0.16 = d	
		*SiSRO2b*				Scaffold	287,017–290,048	NA	
*Citrullus lanatus* watermelon	Trehalose-6-Phosphate Synthetase (TPS)	*ClTPS1*	7	salt and other stresses	Maintaining osmotic pressure, protecting membrane structure, and participating in signal transduction; trehalose upregulated the activities of antioxidant enzymes, such as superoxide dismutase (SOD), ascorbic acid peroxidase (APX), peroxidase (POD), and catalase (CAT); increasing reactive oxygen species (ROS) scavenging capacity	1 (~37 Mb)		0.95 = d	Yuan et al. 2021 [8]
		*ClTPS2*				3 (~32 Mb)		0.26 = p	
		*ClTPS3*				5 (~36 Mb)		0.96 = d	
		*ClTPS4*				6 (~29 Mb)		0.97 = d	
		*ClTPS5*				7 (~32 Mb)		0.06 = d	
		*ClTPS6*				10(~35 Mb)		0.04 = d	
		*ClTPS7*				11 (~30 Mb)		0.95 = d	
Sum								62 d:47 p	

## Data Availability

The data in Table 1 were obtained from the cited publications in the special issue.
